# Epidemiology of Imported Cutaneous Leishmaniasis: A Narrative Review

**DOI:** 10.3390/microorganisms14081774

**Published:** 2026-08-12

**Authors:** Dimitra Bednar, Marcus Cheng, Sophia Salazar, Kyle Seigel, Andrea K. Boggild

**Affiliations:** 1 Division of Dermatology, Department of Medicine, University of Toronto, Toronto, ON M5S 3H2, Canada; 2 Faculty of Arts and Science, University of Toronto, Toronto, ON M5S 3G3, Canada; 3 Division of Infectious Diseases, Department of Medicine, University of Toronto, Toronto, ON M5S 3H2, Canada; 4 Tropical Disease Unit, Toronto General Hospital, University Health Network, Toronto, ON M5G 2C4, Canada; 5 Institute of Medical Science, Temerty Faculty of Medicine, University of Toronto, Toronto, ON M5S 3H2, Canada

**Keywords:** cutaneous leishmaniasis, emerging infectious diseases, imported infections, *Leishmania*, mucosal leishmaniasis

## Abstract

Cutaneous leishmaniasis (CL) is a neglected tropical disease (NTD) traditionally associated with rural poverty in endemic regions but is increasingly encountered among international travelers, migrants, and displaced populations. Climate change, globalization, and mass human mobility have altered the epidemiology of CL, expanding the geographic range of competent vectors and increasing the burden of imported disease in non-endemic countries. Imported cases are of particular public health importance because they may serve as sentinels for autochthonous transmission in regions where vectors and reservoir hosts are present. This narrative review synthesizes surveillance data and epidemiological evidence on imported cutaneous and mucocutaneous leishmaniasis (MCL), with particular emphasis on findings from regional and global surveillance networks. We describe demographic, clinical, and travel-related patterns of imported disease, highlight differences between Old World and New World CL, and discuss implications for surveillance, clinical management, and future research in the context of climate change and global migration.

## 1. Introduction

Cutaneous leishmaniasis (CL) is a parasitic protozoal infection caused by multiple *Leishmania* species and transmitted by infected female phlebotomine sandflies [1,2]. It most commonly manifests as chronic ulcerative lesions but may progress to mucosal leishmaniasis (MCL), particularly in infections caused by the species *Leishmania* (*Viannia*) *braziliensis*, which is considered a type of New World Leishmaniasis found in tropical regions of Central and South America [3,4,5]. Globally, an estimated 0.7–1.2 million new CL cases occur annually, although the true burden is likely underestimated because of incomplete reporting, lack of large-scale regional surveillance efforts, and limited diagnostic access in endemic regions [1,6]. Despite CL often being self-limited and curable, it carries substantial risks of scarring, psychosocial morbidity, and, in cases of MCL, functional and cosmetic sequelae [2,4,5].

Leishmaniasis is classified as a neglected tropical disease (NTD), reflecting chronic underinvestment in research, diagnostics, therapeutics, and surveillance relative to its global burden [1,2]. The neglected status of CL is compounded by its disproportionate impact on populations living in rural poverty, conflict zones, and regions with limited healthcare infrastructures [2,7].

**Figure 1:**
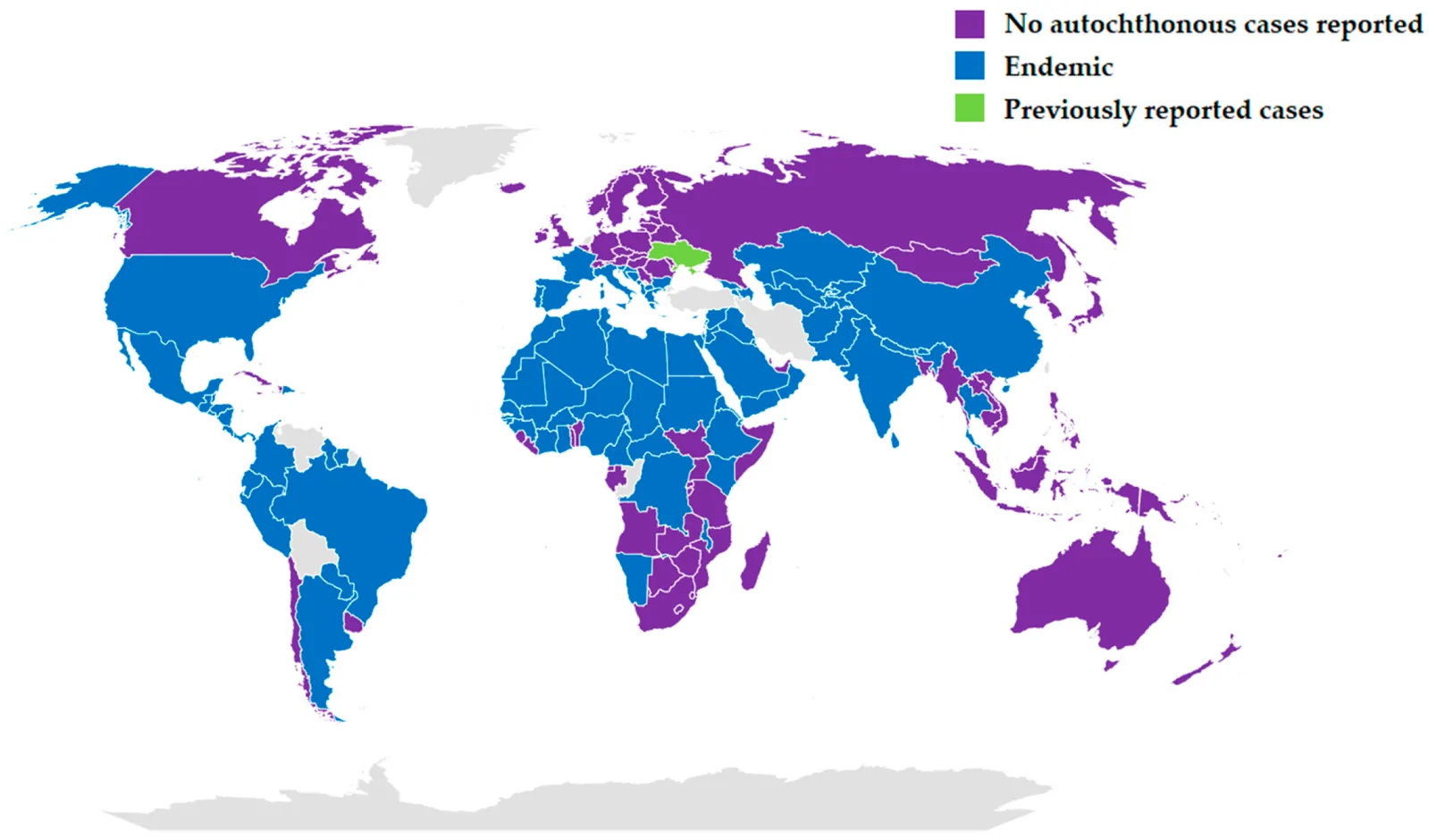
Global status of endemicity of cutaneous leishmaniasis, 2024. Endemicity classifications are based on country-level WHO Global Health Observatory data [14].

While CL is endemic in tropical and subtropical regions (Figure 1), imported cases are increasing in non-endemic countries due to increased international travel, migration, and humanitarian crises originating in areas of endemicity [8,9,10,11]. Climate change has concurrently driven expansion of sandfly vector ranges into temperate regions, including the southern United States and Mediterranean Europe, which raises concern for new foci of endemic transmission [12,13].

Understanding the epidemiology of imported CL is critical for several reasons. First, imported cases may act as sentinels for the potential emergence of native transmission in regions where competent vectors and reservoir hosts exist [12,13]. Second, understanding species-level epidemiology of imported disease has direct implications for clinical management and prognosis, particularly given the risk of mucosal involvement associated with certain New World species, notably strains belonging to the *Viannia* subgenus [3]. Finally, surveillance data on imported CL inform pre-travel counseling, post-travel diagnosis and management, and public health preparedness in non-endemic countries [8,9].

Despite its public health significance, leishmaniasis is not nationally notifiable in several non-endemic countries, including Canada, the United States, and the United Kingdom [12,13,15]. As a result, imported cases are not systematically tracked, and true incidence is likely underestimated. In countries where leishmaniasis is notifiable, particularly in Europe, surveillance practices are variable and under-reporting is common, especially for isolated CL cases [16,17].

Prior to the establishment of large-scale traveler surveillance networks, no global, long-term, or granular datasets describing imported CL and MCL were available. This lack of data limited the ability to characterize demographic and travel-related risk factors, regional patterns of acquisition, and species-level epidemiology among travelers and migrants [8,9]. Surveillance networks—such as GeoSentinel and TropNet, for example—can provide critical data for understanding imported CL and MCL in non-endemic settings and for informing clinical and public health responses. We herein narratively synthesize published surveillance data on imported cases of CL and MCL arising from regional and global networks.

## 2. Methods

This narrative review is based on a synthesis of epidemiological and surveillance data presented in a Public Health Agency of Canada (PHAC) webinar on the epidemiology of imported leishmaniasis [18], with a particular focus on analyses derived from global and regional surveillance consortia. The review integrates descriptive data from published network analyses spanning 1997 to 12 June 2026, including demographic, clinical, travel-related, and microbiological characteristics of travelers and migrants diagnosed with CL and MCL. Additional contextual information on climate change, neglected tropical diseases (NTDs), and vector ecology is incorporated to frame the evolving epidemiology of imported CL.

A literature search was conducted to identify any new surveillance data available since the delivery of the webinar to PHAC. Using combinations of the terms “surveillance”, “travel” and “leishman*”, the databases MEDLINE, Embase, and CENTRAL were searched from inception until 12 June 2026 for relevant surveillance analyses reporting on >25 cases of leishmaniasis. Synthesizing data from networks designed to surveil autochthonous CL and MCL in highly endemic areas, such as SisLeish or PAHO in Latin America, is out of scope for this review [19,20].

Table 1 provides a summary of large surveillance analyses arising from regional and global consortia that describe cases of imported CL and/or MCL.

## 3. Results

### 3.1. Surveillance from the GeoSentinel Surveillance Network and the Regional Consortia CanTravNet and EuroTravNet

GeoSentinel is a global, clinic-based surveillance system for travel-acquired infectious diseases jointly supported by the U.S. Centers for Disease Control and Prevention, the International Society of Travel Medicine, and the Public Health Agency of Canada [27]. The network comprises 68 specialized travel and tropical medicine clinics across 28 countries on six continents. GeoSentinel captures de-identified demographic, travel, and clinical data from ill returned travelers and migrants, linking geographic exposures with syndromic and etiologic diagnoses rendered by expert clinicians using reference-standard diagnostics [27].

The network serves three primary functions: (1) rapid detection of sentinel events, such as emerging or re-emerging infections; (2) surveillance of trends in known travel-related diseases; and (3) analysis of morbidity patterns to inform both clinical care of returned travelers and pre-travel risk counseling. Data collected in GeoSentinel include demographics, travel history including locations and dates, risk activities during travel, purpose of travel, and clinical information regarding the patient’s illness and comorbidities. As of recent estimates, the database includes over 350,000 travelers and more than 450,000 diagnoses acquired in over 250 countries, making it the largest surveillance system of its kind.

CanTravNet is the Canadian consortium of GeoSentinel sites, comprising nine clinics across the country and serving a collective catchment area of nearly half the Canadian population. Through a formal collaboration with PHAC’s Centre for Border and Travel Health, CanTravNet supports national surveillance activities and contributes to federal reporting and public health intelligence. Similarly, EuroTraveNet represents the European sites of the GeoSentinel network, comprising specialized travel and tropical medicine clinics across multiple European countries that collect standardized clinical and epidemiological data on ill returned travelers and migrants. Data are used to monitor trends in imported infectious diseases, detect emerging travel-related health threats, and inform public health responses in Europe.

Early CanTravNet analyses of travel-acquired dermatoses demonstrated that CL consistently ranked among the top ten diagnoses in returned travelers with skin lesions [10]. In a three-year analysis, CL accounted for approximately 3% of dermatologic morbidity, ranking eighth overall in terms of etiologic diagnoses (Table 1) [10]. Travelers with CL tended to have longer trip durations compared to those with other dermatoses (median duration 35 days vs. 8–10 days), and cases were disproportionately associated with migration from countries such as Syria and Afghanistan, highlighting the intersection of travel, migration, and conflict.

Recent surveillance analyses from EuroTravNet describe the importance of imported infections in European travel medicine practices. A 20-year EuroTravNet analysis of 103,739 ill travelers and migrants presenting to 25 European clinics showed that migrants accounted for 43% of cases and VFR travelers for 20%, with both groups increasing over time [21]. Vector-borne diseases represented 12% of all diagnoses and showed a rising trend across the study period [21]. This analysis highlights broader shifts in European travel medicine, where migration, travel patterns, and vector-borne disease exposure increasingly shape the burden of imported infections. Although the total number of leishmaniasis cases is not specifically reported, “*Leishmania*” as an etiologic diagnosis was among the top 20 diagnoses for regions including Central America (n = 41 cases), North Africa (n = 73 cases), Western Europe (n = 94 cases), and the Middle East (n = 40 cases), and VL specifically was causally linked to 3 of the 45 reported deaths in the cohort (Table 1).

A five-year GeoSentinel network-wide analysis of more than 42,000 ill returned travelers identified 264 cases of CL or MCL (Table 1) [9]. Consistent with endemic population studies, there was a marked male predominance, with a male-to-female ratio of approximately 1.9, and a median age of 23 years [9]. Half of cases occurred in tourists, followed by travelers visiting friends and relatives (VFR) and business travelers [9].

Geographic patterns of acquisition were notable. Bolivia, Afghanistan, and Costa Rica emerged as the most common countries of exposure. CL was a leading cause of dermatologic morbidity among travelers to Latin America and the Caribbean, second only to cutaneous larva migrans, and was also highly represented among travelers returning from Europe, the Middle East, North Africa, and South-Central Asia.

To address remaining gaps in the epidemiology of travel-acquired tegumentary leishmaniasis specifically, a comprehensive 20-year GeoSentinel analysis spanning 1997–2017 was conducted (Table 1) [8]. Among nearly 275,000 ill returned travelers, 955 cases of CL or MCL met inclusion criteria after exclusions [8]. Adjusted analyses accounting for expansion of surveillance sites demonstrated sustained importation over time rather than artifactual increases due to improved data capture [8].

Men accounted for nearly two-thirds of all cases and almost three-quarters of MCL cases [8]. While CL affected individuals across a wide age range, MCL occurred exclusively in adolescents and adults [8]. Notably, more than 10% of cases were acquired during trips lasting two weeks or less, underscoring that acquisition of CL is not limited to prolonged or high-risk travel [8].

Tourism was the predominant reason for travel, accounting for over half of CL cases and nearly two-thirds of MCL cases [8]. South and Central America were the leading regions of acquisition, particularly for MCL, with Bolivia and Peru contributing a substantial proportion of cases associated with *L.* (*V.*) *braziliensis* [8]. In contrast, Old World CL was more commonly imported by migrants and VFR travelers, particularly from conflict-affected regions such as Afghanistan and Syria [8].

Species identification was available for approximately 29% of cases [8]. *L.* (*V.*) *braziliensis* was the most frequently identified species among New World imports, while *L. major* predominated among Old World cases, commonly acquired in Israel, Morocco, and Tunisia [8]. Given the proclivity of *L.* (*V.*) *braziliensis* to cause mucosal disease, even after successful treatment of CL, the findings highlight the clinical importance of species-level diagnosis for risk stratification and management.

### 3.2. Surveillance from TropNet Europe, “LeishMan” and +Redivi Network

TropNet Europe is a collaborative clinical consortium that includes a dedicated leishmaniasis surveillance and management arm known as the “LeishMan” network. Comprising specialized European centers, with recent surveillance analyses drawing from up to 15 participating clinics [22], LeishMan functions as a multi-center network that collects standardized case data on ill returned travelers and migrants. By pooling these retrospective clinical data across multiple European countries, the LeishMan surveillance network monitors trends in imported leishmaniasis, which enables the ongoing surveillance of cases, helping to detect emerging travel-related health threats and inform public health responses across the region.

A broad retrospective analysis spanning 2014–2019, which was conducted across 15 European LeishMan network centers, documented records from 1142 cases of leishmaniasis [22]. Among the cohort, CL was the predominant presentation, accounting for 76% of cases, followed by VL at 21% and ML at 3% [22]. Men accounted for over two-thirds of all cases (68%), and the median age at diagnosis was 37 years [22]. The geography of acquisition diverged sharply by clinical form: VL was primarily an autochthonous disease acquired within Europe (88%), whereas CL was predominantly imported from outside Europe (77%) [22]. Among these imported CL cases, 62% originated from the Old World and 38% from the New World [22].

A separate European clinical report evaluated 459 travelers recruited across 10 European LeishMan centers between 2006 and 2019, corresponding to 464 distinct infections acquired across 47 countries [24]. The median age of patients was 30 years, though notably, 21% of infections (99 infections) occurred in children under 16 years of age [24]. Demographic profiles were influenced by travel purpose and destination: tourists returning from the New World (often “backpackers”) were significantly younger than those returning from the Old World (median age 30 vs. 48 years), and military personnel or expatriates acquired infections almost exclusively in the New World [24]. Conversely, migration-related infections were observed only in the Old World, and children were primarily infected while visiting friends and relatives (VFR) or during migration. New World infections showed a strong male predominance (75% male) compared to Old World infections (54.5% male) [24].

Focusing specifically on the Old World burden, an analysis of 206 parasitologically confirmed cases, which were caused specifically by *Leishmania major* and *L. tropica* within the LeishMan network between 2013 and 2019, echoed the epidemiological divide driven by the patients’ reasons for travel [25]. Among the 206 cases, 131 were identified as *L. tropica* and 75 as *L. major* [25]. The burden of *L. tropica* fell overwhelmingly on migrants, who accounted for 80% of these infections [25]. Furthermore, nearly half (48%) of all patients infected with *L. tropica* were children under the age of 15 [25]. In contrast, the majority of *L. major* infections (53%) occurred in travelers who had been visiting friends and relatives (VFR) in endemic regions [25].

The +Redivi network comprises 25 specialized and non-specialized travel medicine centers across nine regions of Spain. New cases of imported infections are registered within a common online database, where patients are classified as migrants, VFR migrants (returning from their country of birth after visiting friends and relatives), VFR travelers (returning from a first-degree relative’s country of birth), and travelers. The same traveler may account for more than one entry within the network if they have been on different trips and have been diagnosed with an imported disease; in other words, entries in +Redivi are per travel-related illness rather than per individual. A recent analysis of dermatoses in the network demonstrated that throughout the period from October 2009 to December 2024, a total of 35,632 individuals were registered within the network, of whom 3437 presented with a cutaneous syndrome. Over half (53.6%) of the population was female, and travelers predominantly represented the group (62.4%), followed by migrants (22.4%) and VFRs (15.2%). The median age of the group was 36.1 years, and the travel duration was a median of 30 days for VFRs and 17 days for travelers [26]. Different groups also exhibited different times to consultation, with the median for migrants being 1059 days, compared to medians of 34 days (VFRs) and 11 days (travelers) [26]. The most frequently reported exposures arose from Thailand (9.4%), Brazil (4.8%), Indonesia (4.5%), Senegal (4.4%), and Mexico (4%) [26].

During the study period, 60 CL cases were reported in total, constituting 1.75% of all patients who consulted the network for a cutaneous syndrome [26]. More than half of CL cases occurred in travelers (n = 32), followed by a quarter in VFRs (n = 16) and a fifth of cases in migrants (n = 12) [26]. However, proportionately, VFRs were overrepresented among cases of CL compared to both migrants and travelers, at 3% of all cutaneous diagnoses vs. 1.6% and 1.5%, respectively [26].

## 4. Discussion

### 4.1. Climate Change, Vector Ecology, and Emerging Endemicity

The geographic range of phlebotomine sandflies has expanded in response to rising temperatures and altered precipitation patterns. Ecological niche modeling studies have demonstrated northward expansion of competent vectors and rodent reservoir hosts, particularly in North America. While vector distributions remain more geographically constrained, reservoir species capable of sustaining transmission are often more widely distributed, creating ecological conditions conducive to the establishment of new endemic foci.

In the United States, modeling studies have suggested the potential for leishmaniasis surveillance north of Texas as vector and reservoir ranges continue to expand [12]. Autochthonous cases of CL have been reported from Texas, Oklahoma, Arizona, and South Dakota [28,29]. Similar concerns exist in southern Europe, where autochthonous transmission has already been documented in some Mediterranean countries. In recent years, significant autochthonous transmission leading to human cases of CL within Mediterranean nations was reported from France [23], Italy [30,31], Spain [32], Portugal [33], and Greece [34]. These trends underscore the importance of robust surveillance systems capable of detecting imported cases that may herald local transmission in countries that were previously and exclusively importers of cases.

### 4.2. Implications for Clinical Practice and Public Health

Imported CL represents an increasingly important diagnosis for clinicians caring for returned travelers and migrants. Awareness that CL may be acquired during short trips, that tourists constitute a substantial proportion of cases, and that certain regions confer high risk for mucosal disease is essential for timely diagnosis, prompt initiation of effective therapy, and appropriate follow-up [8]. Moreover, discussion of CL as a potentially morbid travel-related disease should be addressed in the pre-travel medical consultation setting for travelers going to high-risk countries and regions. Given the broad differential diagnosis of ulcerative lesions in returning travelers, and given that differentiating locally acquired from true travel acquisition in areas where both are possible may be challenging [35], continued diagnostic innovation towards platforms that are accessible, reliable, accurate, reliant on specimens that are easy to collect at the bedside [36], and able to resolve the causative organism down to the species level should be encouraged [37].

From a public health perspective, imported cases serve as early warning signals for potential local transmission risk in regions experiencing vector expansion or already maintaining populations of competent phlebotomine vectors [13,19]. Where competent vectors exist, importation of cases among humans has the potential to establish transmission among reservoir hosts (e.g., dogs) and vice versa. As such, coordination of public health surveillance and control efforts in non-endemic regions subject to importation via travelers should necessarily involve those with entomological, veterinary, and One Health expertise.

### 4.3. Limitations of Surveillance Data

GeoSentinel provides unparalleled longitudinal and geographic coverage of travel-associated CL and MCL but still has several limitations that warrant consideration: (1) data are derived from specialized clinics and may not be generalizable to all travelers or migrants with leishmaniasis; (2) pediatric populations are under-represented; (3) detailed diagnostic methods and species identification techniques are not uniformly captured; and (4) it lacks granular data on treatment regimens and long-term outcomes needed to guide clinical protocols facing specific diseases.

Similarly, surveillance data arising from TropNet Europe are limited by the sentinel clinic-based design. These datasets capture ill travelers and migrants who present to participating specialist centers and therefore cannot estimate true population incidence [21]. Observed trends may be affected by changes in site participation, referral patterns, travel and migration patterns, and diagnostic capacity [21]. Since these surveillance systems are designed to monitor broader travel-related morbidity rather than CL/MCL specifically, they may also lack detailed species-level, treatment and long-term outcome data needed to inform disease-specific clinical protocols [21].

Given overlapping recruitment and inclusion periods across the various surveillance analyses and by different networks, individual cases of leishmaniasis are almost certainly represented in several analyses. As such, it is challenging to understand the absolute number of unique cases imported to various regions over time.

### 4.4. Future Directions

Given its status as an NTD, enhanced investment in surveillance, diagnostics, and research is urgently needed to address the epidemiology of CL, which is rapidly evolving due to climate changes. Noting the limitations above, future surveillance efforts should prioritize standardized reporting across participating sites to improve the consistency, comparability and overall quality of imported leishmaniasis data.

Expanding the use of molecular diagnostics and centralized species typing would strengthen comparisons across regions and facilitate earlier recognition of evolving transmission patterns. In parallel, integrating imported case surveillance data with entomological and ecological monitoring may improve early detection of emerging endemicity.

Additional pediatric-focused surveillance and robust outcome studies are also needed, as children remain underreported and may experience differences in exposure patterns, clinical presentation, treatment tolerability and long-term outcomes. Improved clinical awareness and clearer referral pathways for molecular confirmation and species typing would further strengthen coordinated public health responses to imported leishmaniasis. Moreover, future directions should emphasize raising public awareness about travel-acquired CL—particularly in countries of Europe and North America, which supply heavy travel volumes to areas of MCL risk—due to its personal health impact and public health significance.

In 2025, Tropistry was an attempt in the field to improve large surveillance networks [38]. Planned for implementation across European sites, it aims to improve generalizability by decentralizing data collection, mainly through equipping frontline hospitals, in addition to specialized laboratories, with active clinical decision support and expert access [18]. Concurrently, Tropistry’s Case Report Forms separate different diseases into distinct modules, thus enforcing standardized logging of exact diagnostic methods, species identification, and longitudinal treatment outcomes [18]. Successful implementation and sustainability will address many of the aforementioned challenges limiting the generalizability, accuracy, and precision of historic surveillance networks.

## 5. Conclusions

Cutaneous leishmaniasis is no longer confined to endemic regions but is an emerging global health concern driven by climate change, globalization, and human mobility. Surveillance data from regional and global consortia demonstrate distinct demographic and travel-related patterns of imported disease, with important implications for clinical care and public health preparedness. Strengthening surveillance systems that embed epidemiological and biological data (particularly those underpinned by molecular diagnostics) while maintaining clinical vigilance is critical to preventing, detecting, and managing this neglected yet increasingly relevant infection in mobile populations.

## Figures and Tables

**Table 1:** Included Surveillance Studies Informing Narrative Review of Imported CL/MCL Cases.

Study	Data Source	Years	Population	CL/MCL Cases	Key Findings
Stevens et al. 2015 [10]	CanTravNet	2009–2012	Returned Canadian travelers and immigrants with travel-related dermatoses	36 CL	CL was regularly encountered in Canadian travel and immigrant health clinics and was associated with diverse travel categories, including tourism, immigration/refugee movement, VFR (visiting friends and relatives) travel, and volunteer- or aid-work related travel.
Leder et al. 2013 [9]	GeoSentinel	2007–2011	Ill returned travelers presenting to GeoSentinel sites	264 CL/MCL	CL/MCL was a significant cause of travel-related dermatologic illness in an international surveillance network. Bolivia, Afghanistan, and Costa Rica were identified as major exposure locations, emphasizing destination-specific risk patterns and the global distribution of CL/MCL.
Boggild et al. 2019 [8]	GeoSentinel	1997–2017	International travelers and migrants diagnosed with travel-acquired CL or MCL	916 CL and 40 MCL	Largest multinational analysis of imported CL/MCL and showed that ~10% of infections occurred after trips of ≤2 weeks. Bolivia and Costa Rica were major acquisition sites among travelers, whereas Syria and Afghanistan predominated among migrants. *L.* (*V.*) *braziliensis* was the most identified species, and MCL was strongly associated with tourism-related travel to Bolivia.
Lemieux et al. 2022 [15]	Canadian reference centre	10 years	Travelers and migrants diagnosed with laboratory-confirmed CL in a Canadian tropical disease reference centre	48 CL	Increasing imported CL cases in Canada, with most infections acquired in Central and South America. The study highlighted prolonged diagnostic delays and frequent need for systemic therapy, particularly liposomal amphotericin B, underscoring ongoing challenges in recognition and management in non-endemic settings.
Grobusch et al. 2021 [21]	EuroTravNet	1998–2018	Ill travelers and migrants in 25 European clinics	At least 248 CL/MCL/VL (visceral leishmaniasis)	An etiologic diagnosis of “*Leishmania*” was within the top 20 regional diagnoses for Central America (n = 41), North Africa (n = 73), Western Europe (n = 94), and the Middle East (n = 40). VL was causally related to 3 deaths (of 45 total deaths in the cohort). The total number of cases of CL/MCL/VL is not reported.
Van de Auwera et al. 2022 [22]	European LeishMan Network	2014–2019	Patients diagnosed at 15 centers across 11 European countries	865 CL and 33 MCL	Of imported European CL cases, 62% were Old World and 32% were New World. Old World CL was commonly observed in migrants from Syria and Afghanistan, whereas New World CL was mainly observed among tourists returning from Peru, Costa Rica, Brazil and Bolivia.
Pasquier et al. 2022 [23]	France National and Regional Surveillance Database	1998–2020	Leishmaniasis patients from French National Reference Centre for Leishmaniasis (over 200 sources)	91 CL and 15 MCL	Stable endemic transmission in overseas territories alongside lower but persistent case numbers in metropolitan France. Showed coexistence of autochthonous and imported cases, with clear geographic variation in incidence and species distribution.
Guery et al. 2021 [24]	European LeishMan Network	2006–2019	Patients diagnosed with leishmaniasis collected across 10 European LeishMan centres	459 CL	Imported tegumentary leishmaniasis cases documented across the European LeishMan centres were strongly associated with distinct, geography-dependent demographic and travel categories, including New World tourism and military deployment, alongside Old World migration and VFR travel.
Glans et al. 2022 [25]	European LeishMan Network	2013–2019	Patients diagnosed with leishmaniasis within the LeishMan network	206 CL	Old World CL cases within the network demonstrated a species-specific epidemiological divide, with *L. tropica* predominantly burdening migrant populations and young children, while *L. major* was mainly associated with VFR travel.
Gayoso-Cantero et al. 2026 [26]	+Redivi Network	2009–2024	Patients with a cutaneous syndrome as reasons for consultation within the +Redivi Spanish national network (25 centres)	60 CL	CL cases were distributed over three groups: migrants, VFR, and travelers. Among the three, CL cases within the network were significantly associated with the traveler group (53.3% of all CL cases).

CL—cutaneous leishmaniasis; MCL—mucocutaneous leishmaniasis; VL—visceral leishmaniasis.

## Data Availability

No new data were created or analyzed in this study. Data sharing is not applicable to this article.
